# Effect of multidisciplinary team (MDT) centred on pregnant women with pulmonary hypertension on treatment and outcomes of pregnancy

**DOI:** 10.1186/s12890-023-02355-1

**Published:** 2023-02-11

**Authors:** Wenjie Chen, Jun Luo, Jingyuan Chen, Yusi Chen, Zilu Li, Haihua Qiu, Jiang Li

**Affiliations:** Department of Cardiovascular Medicine, The Second Xiangya Hospital of Central South University, No. 139 Middle Renmin Road, Furong District, Changsha City, 410011 Hunan Province China

**Keywords:** Multidisciplinary team, Pulmonary hypertension, Pregnant outcomes, Targeted therapy

## Abstract

**Background:**

The importance of multidisciplinary team (MDT) centred on pregnant women with pulmonary hypertension (PH) has been highlighted. However, rare studies have explored its effects on pregnancy outcomes. This study seeks to investigate whether and how the MDT has an effect on the treatment and outcomes of PH pregnant women.

**Methods:**

A pre- and post-intervention study was conducted based on an interrupted time series design to compare the treatment and outcomes of patients with PH before (pre-MDT) and after (post-MDT) implementation of the MDT. PH was defined as pulmonary artery systolic pressure (sPAP) ≥ 35 mmHg measured by echocardiography or right heart catheterization and sPAP at 35–60 mmHg and over 60 mmHg was defined as mild and severe PH, respectively. All results were analyzed by T-tests, Chi square tests or Fisher exact test and two-sided *p* value < 0.05 was set to be statistically significant.

**Results:**

149 pregnancies were found in 143 women with PH. Overall, 46 pregnancies were elective abortions, remaining 49 and 54 pregnancies completing delivery in the pre-MDT group and post-MDT group, respectively. Five (10.2%) mother and seven (8.6%) neonatal died in the former, while no maternal deaths but 1.9% neonatal death occurred in the latter. In subgroup analysis, maternal and fetal/neonatal complications were higher in patients with severe PH and World Health Organization functional class (WHO FC) III/IV and all maternal deaths occurred in class III/IV women. In pre-MDT and post-MDT groups, there were 8 and 22 pregnant women receiving the pulmonary-specific therapy and completing delivery, respectively. The percentage of heart failure and urgent cesarean of pre-MDT group was higher than the post-MDT group (30.6% vs. 12.9%, *p* = 0.02; 40.8% vs. 14.8%, *p* = 0.01, respectively).

**Conclusion:**

Implementing the MDT decreased the rate of urgent caesarean section and heart failure in patients with PH and no maternal deaths occurred in the post-MDT group. Pregnant women with severe PH and WHO FC III/IV might have a poor prognosis, whereas the use of pulmonary-specific therapy might improve outcomes of pregnancy.

## Introduction

Pulmonary hypertension (PH) is a pathophysiological disorder characterised by an increase in pulmonary arterial pressure (PAP), resulting in right heart failure and death [1]. In 2018, the 6th World Symposium on Pulmonary Hypertension (WSPH) updated the PH classification and divided PH into five groups in accordance with their pathogenesis and treatment strategies: (1) pulmonary arterial hypertension (PAH; group 1 PH); (2) PH arising from left heart disease (LHD-PH; group 2 PH); (3) PH caused by lung diseases and/or hypoxia (group 3 PH); (4) PH due to pulmonary artery obstructions (group 4 PH); and (5) PH with unclear and/or multifactorial mechanisms (group 5 PH) [2].

Pregnancy with any type of PH is a potentially life-threatening combination as there are limited therapies and poor pregnancy outcomes are frequent, including an alarming maternal mortality rate of 30–56% [3, 4]. Thus, a consensus has been reached that women with PH (especially PAH) should adopt effective contraception and undergo early termination of accidental pregnancy [5–8]. However, the risks associated with pregnancy termination are higher in patients with PH than in the general population, and pregnancy termination is recommended at experienced PH centres [9].

Over the last decade, PAH-specific therapies, consisting of prostaglandin analogue (e.g., epoprostenol) and phosphodiesterase 5 inhibitors (e.g., sildenafil), have been used for the treatment of China’s pregnant women with PAH [10, 11]. However, recent studies and a meta-analysis suggested that the maternal mortality rate remains between 12 and 20% in pregnant PH patients, and most maternal deaths occur in the early postpartum period [11–13]. Accordingly, improving the perinatal outcomes in women with PH remains challenging and usually involves the combination of multiple disciplines. To better care for pregnant women with PH, in December 2012, a multidisciplinary term (MDT) centred on women with PH was established at our hospital. Although the importance of the MDT has been already highlighted by guidelines and clinic experience, whether and how the MDT approach affects treatment and outcomes of pregnant women with PH remains unclear [14, 15].

To verify the effectiveness of the MDT model, a longitudinal analysis was conducted on the characteristics, treatment, and outcomes of patients treated by our MDT, and patients treated before and after the establishment of this team were compared.

## Methods

This retrospective study obtained approval from the review board of the Second Xiangya Hospital of Central South University. The electronic medical records of 143 pregnant women with PH (six had two pregnancies) admitted into our hospital from January 2004 to June 2020 were reviewed. The PAP was evaluated using either echocardiography or right heart catheterisation (RHC). RHC has been recognised as the “gold standard” for the diagnosis of PH (mPAP ≥ 25 mmHg). Echocardiography is a non-invasive method that has been confirmed as an accurate method of evaluating PAP [16]. In this study, the patients with a pulmonary arterial systolic pressure (sPAP) of higher than 35 mmHg were considered to have PH, and the patients with a sPAP of 35–60 mmHg and > 60 mmHg were defined as mild and severe PH, respectively. Furthermore, the baseline characteristics, diagnosis and treatment plan, maternal and infant outcomes, and follow-up information of all patients were extracted.

**Fig. 1:**
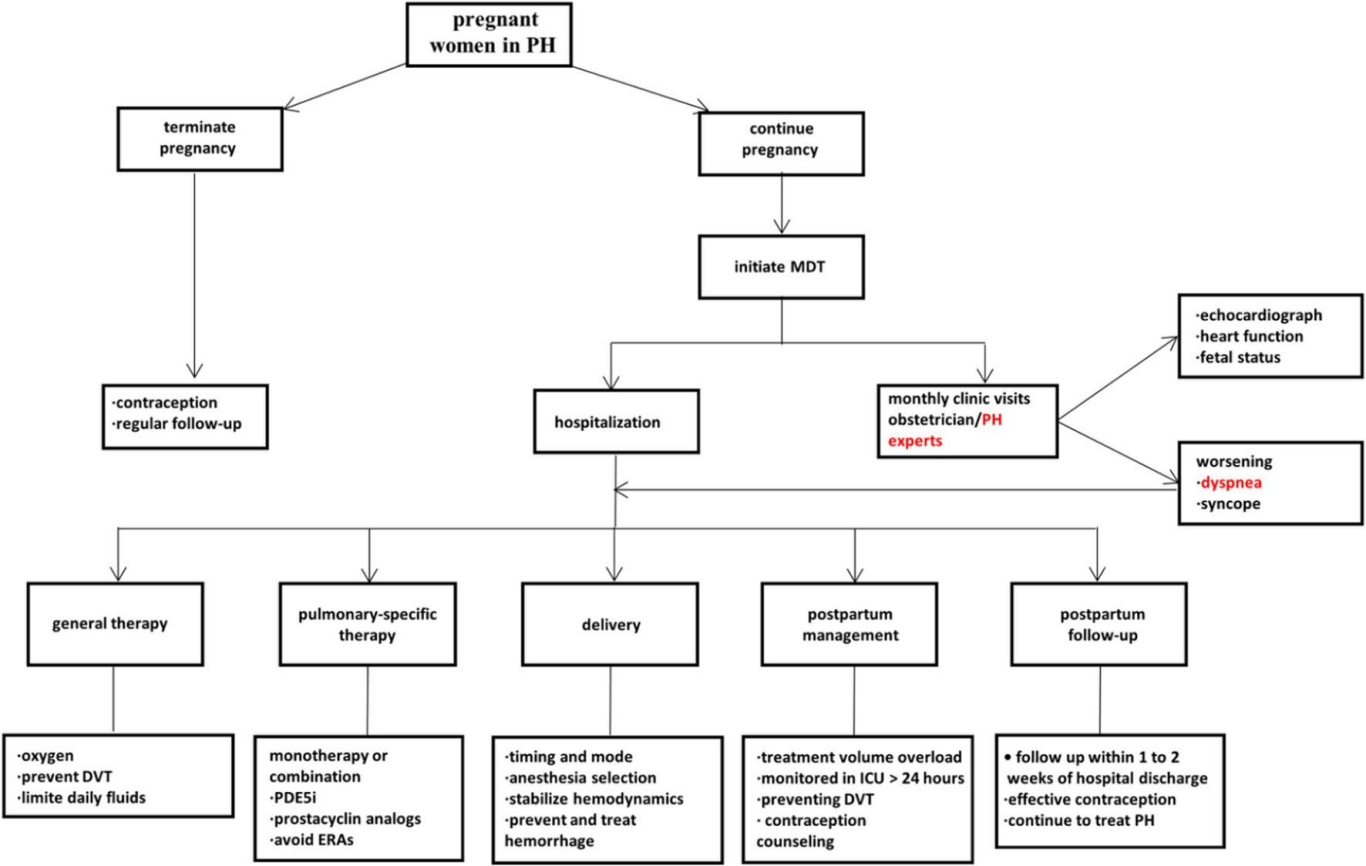
The treatment strategy of multidisciplinary team central on pregnant women with pulmonary hypertension. DVT: deep vein thrombosis; ERAs: endothelin receptor antagonists; ICU: Intensive care unit; MDT: multidisciplinary team; PDE5i: phosphodiesterase 5 inhibitors; PH:pulmonary hypertension

To better care for pregnant women with PH, the MDT centred on pregnant women with PH was established at our hospital in December 2012. One month (from December 1 to 30, 2012) was set as a “run-in” period, and information regarding MDT was disseminated at our hospital. Thus, the data fell into pre-MDT (01/2004–11/2012) and post-MDT (1/2013–6/2020) groups. The MDT consisted of an obstetrician, respiratory specialist, cardiologist, anaesthesiologist, rheumatic immunologist, cardiac surgeon, neonatologist, and critical care specialist. Figure 1 illustrates the treatment strategy of the MDT.

The aim of this study was to conduct a retrospective study and evaluate the effect of the introduction of a MDT program on treatment and outcomes in pregnant women with PH, whereas some unmeasured confounders (e.g., the advance of medicine and technology) could not be avoided.

The following data were collected by our team. The basic information collected included age and World Health Organization functional class (WHO FC). The characteristics of PH involved aetiology, echocardiography, and PAP. The obstetric characteristics covered parity, time and mode of delivery, anaesthetic management, as well as baby weight. Patients were followed up by phone for up to 3 months, if possible.

All statistical analyses were conducted using IBM SPSS (Version 25) software. Continuous variables were expressed as mean ± standard deviation or median with range. Pre- and post-MDT patients were compared through t-tests and Wilcoxon rank sum texts for continuous variables, as well as through Chi square tests and Fisher exact tests for categorical variables. The Mann–Whitney test was used to analyse the ordinal variables. A subgroup analysis of severity of PH and level of WHO FC was conducted to explore their effects on maternal and foetal/neonatal outcomes. For all analyses, a two-sided *p* value < 0.05 indicated statistical significance.

## Results

### Analysis of baseline characteristics in two groups

149 pregnancies occurred in 143 women with PH, and 46 pregnancies were elective abortions that were mostly performed before 20 weeks of gestation, thus leaving 49 pregnancies and 54 pregnancies completing delivery for the statistical analysis in the pre-MDT group and in the post-MDT group, respectively. The rate of therapeutic abortion in the pre-MDT group was significantly lower than that of the post-MDT group (19.7% vs. 38.6%; *p* = 0.01). Table 1 lists the characteristics of the pregnant women with PH completing delivery.

**Table 1:** Baseline characteristics of pregnant women with pulmonary hypertension in pre-MDT and post-MDT groups

	Pre-MDT (n = 49)			Post-MDT (n = 54)		p value
	n. or median	%/range		n. or median	%/range	
Demographics and obstetric characteristics						
Age, years (median,range)	25	(20,44)		28	(18,40)	0.01
Nulliparous	37	75.5%		32	59.3%	NS
Weeks of delivery (median,range)	37	(31, 42)		36	(27, 40)	NS
Regular follow-up	26	53.0%		43	79.6%	< 0.01
Classification and etiology of PH						NS
WHO group 1						
IPAH	1	2.0%		3	5.6%	
CTD-PAH	2	4.1%		1	1.9%	
CHD-PAH	30	61.2%		33	61.2%	
WHO group 2						
Valvular disease	14	28.6%		12	22.2%	
WHO group 5						
Single ventricle	2	4.1%		3	5.5%	
Double outlet of RV	0	0.0%		1	1.8%	
Aortic-pulmonary artery collateral circulation	0	0.0%		1	1.8%	
Severity of PH						
Mild	24	49.0%		20	37.0%	NS
Severe	25	51.0%		34	63.0%	
WHO FC						NS
I	6	12.2%		5	9.3%	
II	25	51.0%		25	46.3%	
III	9	18.4%		20	37.0%	
IV	9	18.4%		4	7.4%	

*p* < 0.05 represents a significant difference; Maternal age and weeks of delivery were analyzed by Wilcoxon rank sum text; Other data were analyzed through chi-square test and Fisher exact test; n number of pregnancies; % percentage; CHD congenital heart disease; CTD connective tissue disease; IPAH idiopathic pulmonary arterial hypertension; MDT multidisciplinary team; NS no significance; PH pulmonary hypertension; RV right heart; WHO FC World health Organization function class

A significant difference in the median age of pregnant women between the pre-MDT group and the post-MDT group was identified (25; range [20,44] vs. 28; range [18,40], *p* = 0.01). The rate of regular follow-up for the pregnant women with PH increased by around 50%, from 53.0% of the pre-MDT to 79.6% of the post-MDT in our hospital (*p* < 0.005). However, for median gestational age at delivery, no significant difference was found in the two groups (37, range [31,42] vs. 36, range [27,40], *p* = 0.275). Most women with PH were diagnosed as congenital heart disease-associated PAH (CHD-PAH) in both pre-MDT and post-MDT groups (n = 30/49, 61.2% vs. n = 33/54, 61.2%). Both pre-MDT and post-MDT groups had similar numbers and percentage of severe PH and WHO FC.

### Comparison of management in two groups

Table 2 lists the detail of management for women with PH in pre-MDT and post-MDT groups. 33 and 37 pregnant women with group 1 PH (PAH) were indicated to undergo PAH-specific therapy during pregnancy. In the pre-MDT group, only 8/33 (24.2%) pregnant women with PAH underwent PAH-specific therapy before delivery and majority (n = 8/9) of the above only received monotherapy. After the MDT was established, 22/37 (59.5%) received PAH-specific therapy, and 10 of 22 (45.5%) underwent combination therapy (e.g., sildenafil or tadalafil combined with iloprost or treprostinil).

**Table 2:** Treatment of pregnant women with pulmonary hypertension in pre- MDT and post-MDT groups

	Pre-MDT (n = 49)			Post-MDT (n = 54)		p value
	n	%		n	%	
Pulmonary-specific therapy	8	24.2%		222	59.5%	< 0.01
Monotherapy	7			12		
Combination therapy	1			10		
Oxygen therapy	34	69.4%		32	59.3%	NS
Diuretic	20	40.8%		21	38.9%	NS
Digoxin or Cedilan	18	36.9%		16	29.6%	NS
Mode of delivery^a^						NS
Vaginal	2	4.2%		1	1.9%	
Cesarean section	46	95.8%		53	98.1%	
Anesthesia^a,b^						< 0.01
General anesthesia	18	39.1%		38	71.7%	
Neuraxial^c^	28	60.9%		15	28.3%	
Monitored in ICU^d^	20	40.8%		43	79.6%	< 0.01

***p*** < 0.05 represents a significant difference; n number of pregnancies; % percentage; ^a^1 pregnant delivery and anesthesia information missing in the pre-MDT group; ^b^ Only including cesarean delivery cases: pre-MDT (n = 46), post-MDT (n = 53); ^c^ Neuraxial: epidural or spinal or spinal-epidural anesthesia; ^d^ pregnant women after delivery were admitted to ICU monitored at least 24 h. MDT multidisciplinary team; ICU intensive care unit; NS no significance

Except for a patient (in the pre-MDT group) missing delivery information, detailed birth records were collected for 102 patients. Both groups of pregnant women delivered primarily through cesarean section (95.8% vs. 98.1%). However, there was significant difference in the two groups in anesthesia. 39.1% of pregnancies delivered through cesarean section under general anesthesia, and 60.9% (n = 28/46) delivered under chosen epidural or spinal or epidural-spinal anesthesia in the pre-MDT group, whereas general anesthesia was found as the major anesthesia method (71.7%) of cesarean in the post-MDT group. Furthermore, the pre-MDT group, more pregnant women with PH (n = 20/48, 41.6%) who had an unstable hemodynamics required emergent cesarean section for delivery compared with 14.8% (n = 8/54) patients in the post-MDT group (p = 0.001).

### Maternal and fetal/neonatal outcomes in two groups and subgroup analysis

Table 3 lists the maternal and fetal/neonatal outcomes. Five pregnant women died, and all of them (n = 5/49, 10.2%) occurred in the pre-MDT groups within 2 weeks after delivery and were diagnosed as CHD-PAH. To be specific, two women had patent ductus arteriosus (PDA), one woman had atrial septal defect (ASD), and two women had ventricular septal defect (VSD). Table 4 lists the characteristics of dead pregnant women. In the post-MDT group, there was no death, and the incidence of heart failure also significantly decreased from 30.6% of pre-MDT to 12.9% (*p* = 0.02). However, the preterm delivery significantly increased in the post-MDT group (34.7% vs. 55.6%, *p* = 0.03).

**Table 3:** Maternal and fetal/neonatal complications in pre-MDT and post-MDT groups

Obstetrical complications^a^	Pre-MDT (n = 49)		Post-MDT (n = 54)		*P* value
	n	%	n	%	
Pre-eclampsia	4	8.2%	4	7.4%	NS
Premature rupture of membranes	4	8.2%	5	9.3%	NS
Postpartum haemorrhage^b^	2	4.1%	2	3.7%	NS
Heart failure	15	30.6%	7	12.9%	0.02
Maternal death	5	10.2%	0	0.0%	0.02

Fetal/neonatal complications^c^	Pre-MDT (n = 49)		Post-MDT (n = 52)		
	n/mean	%/SD	n/mean	%/SD	
Birth weight (g), mean ± SD^d^	2469	652	2481	679	NS
Prematurity	17	34.7%	30	55.6%	0.03
Low birth weight^d+e^	21	43.7%	26	53.1%	NS
Neonatal death	7	14.2%	1	1.9%	0.02
IUGR^f^	16	32.6%	8	15.4%	0.04
Naonatal anormaly	2	4.1%	3	5.8%	NS

*p* < 0.05 represents a significant difference; n number of pregnancies or neonatus; % percentage; ^a^ 49 women in the pre-MDT group and 53 women in the post-MDT group; ^b^ pregnant women with cesarean section lose more than 1000 ml of blood or with vaginal delivery than 500 ml; ^d^ one and three baby weight lost in the pre-MDT group and the post-MDT group, respectively; ^e^ was defined as baby weight < 2500 g; ^f^ IUGR defined as birthweight 10th percentile for gestational week at delivery; IUGR intrauterine growth restriction; MDT multidisciplinary team; NS no significance; SD standard deviation

**Table4:** Characterization of maternal mortality in pulmonary hypertension

Patients	Etiology	sPAP (mmHg)	Delivery timing (weeks)	Delivery mode	Anesthesia	Rescue therapy	Timing after delivery	Cause of death
1	PDA	105	34	Urgent cesarean	Epidural-	dopamine + adrenaline + ventilator	3 days	Severe preeclampsia, heart failure associated with multiple organ failure
2	PDA^a^	89	35	Urgent cesarean	General-	nitric oxide + epinephrine + dopamine isoproterenol + norepinephrineventilator + CPR	10 days	Persistent hypoxemia, circulatory collapse and hypoxic encephalopathy
3	VSD	57	33	Urgent cesarean	General-	epinephrine + dopamine + ventilator + CPR	within 24 h	Multiple organ failure, postpartum hemorrhage(1500 ml)
4	ASD	87	38	Urgent cesarean	General-	dopamine + epinephrine + dopamine + ventilator	10 days	Severe preeclampsia, circulatory collapse, hypoxic encephalopathy
5	VSD^b^	84	33	Planned cesarean	Epidural-	Nebulized iloprost + Nitric oxide + defibrillation	2 days	Sudden ventricular fibrillation with RV systolic pressure as high as 132 mmHg

^a^left-to-right shunting developed into right-to-left shunting or bidirectional shunting(Eisenmenger syndrome;^b^postoperative pulmonary arterial hypertension; ASD atrial septal defect; PDA patent ductus arteriosus; VSD ventricular septal defect; sPAP pulmonary artery systolic pressure; CPR cardiopulmonary resuscitation

**Fig. 2:**
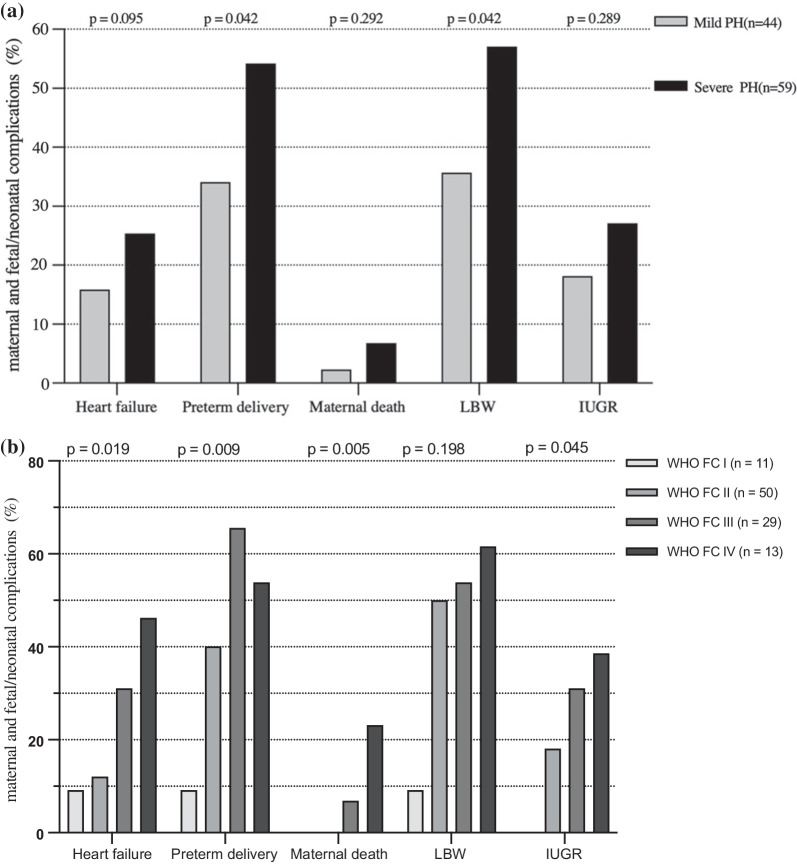
**a** Maternal and fetal/neonatal complications in different severity of PH. PH: pulmonary hypertension; LBW: lower baby weight; IUGR: intrauterine growth restriction.**b** Maternal and fetal/neonatal complications in different WHO FC. WHO FC: World Health Organization functional classification; LBW: lower baby weight; IUGR: intrauterine growth restriction

49 and 52 live births were found in pre-MDT and post-MDT groups, respectively, and there was a twin pregnancy in each group. The neonatal mortality rate of 14.2% (n = 7/49) was significantly higher in the pre-MDT group than the mortality rate of 1.9% (n = 1/52) in the post-MDT group (*p* = 0.03). Moreover, there were also higher percentage of intrauterine growth restriction (IUGR) in pre-MDT (32.6% vs. 15.4%, *p* = 0. 02). Figure 2a and b indicate that the subgroup analyses were conducted by severity of PH and level WHO FC, respectively. Maternal and fetal/neonatal complications were found to be higher in the severe PH group, and a significant difference was found in preterm delivery and low birth weight infants. The similar results were achieved in the subgroup of WHO FC. All maternal deaths were found in WHO FC III/IV women, in which the incidence of heart failure was significantly higher than that of pregnancy with WHO FC I/II.

## Discussion

In the management of pregnant women with PH, especially PAH, the importance of multidisciplinary collaboration has been repeatedly stressed by expert consensus and clinicians; however, there are very few comparative studies on the role and value of the MDT. Although the maternal mortality rate was as high as 10.2% in the pre-MDT group in this study, there were no deaths after the implementation of the MDT at our hospital. As demonstrated by the above result, the MDT contributed to the reduction of maternal mortality, consistent with existing reports [15]. Our MDT was formed in December 2012, consisting of an obstetrician, PH specialist, cardiologist, anaesthesiologist, cardiac surgeon, neonatologist, and critical care specialist, aiming to streamline treatments and improve maternofoetal outcomes in pregnant women with PH (Fig. 1). The MDT focused on and evaluated the risks and benefits of continuing or terminating pregnancy in terms of the life of the mother and foetus, and making decisions timely. To determine whether and how having an MDT centred on pregnant women with PH can affect maternal treatment and outcomes, the first larger group was presented based on the comparison of the characteristics, treatments, and outcomes of PH patients before and after the introduction of the MDT.

### Therapeutic abortion and postpartum care

Changes in the cardiovascular system during pregnancy and delivery lead to PH worsening in women with PH since their compensatory mechanisms for the changes may be disabled [17]. Plasma volume and cardiac output (CO) will start to increase early in pregnancy and reach the peak around 32 gestational weeks at values 40–50% above pre-pregnancy values [18, 19]. Moreover, the second trimester has been generally confirmed as the earliest period of deterioration of heart function for pregnant women with PH [20]. Accordingly, consensus guidelines have recommended women with PH to avoid pregnancy, and the first trimester is considered the safest time to terminate a pregnancy [9, 14]. After the MDT was established, the percentage of therapeutic abortions increased from 19.7% of pre-MDT to 38.4% (*p* = 0.014), accompanied by a decline in the rate of maternal mortality and cardiac complications. Notably, in PH patients, pregnancy termination may lead to pregnancy death and should be performed at an experienced centre [5, 21]. In women with PH, most pregnancy deaths occur in the early postpartum period [11–13], largely caused by right heart (RV) failure and cardiovascular collapse [12, 22, 23]. In this study, four women died of RV failure 2 weeks after delivery, and one died suddenly on the third day after being discharged from hospital. As a result, postpartum management was particularly strengthened after the MDT was established. In general, pregnant women with PH, particularly those with severe PH and WHO FC III/IV, were carefully monitored in an intensive care unit (ICU) at least 24 h after delivery or after haemodynamics became stable.

## Management of pregnant women with PH

### Regular follow-up and general therapy

All women with PH who insist on continuing their pregnancy are required to visit PH experts and obstetricians monthly or weekly for evaluation of pregnancy heart function and PAP by echocardiography. After the implementation of the MDT, the rate of regular clinic visits significantly increased to 79.6% associated with a decline in maternal complications. In addition, pregnant patients with signs of worsening RV function, WHO FC, or PAP were admitted to hospital and received supported therapy including oxygen, diuretics, and digoxin, if indicated. Figure 1 illustrates the treatment steps for women with PH after the implementation of the MDT at our PH centre. Furthermore, severity of PH and WHO FC might be correlated with adverse effects on the maternal and foetal/neonatal outcomes, as presented in Fig. 2a, b. Thus, PAP/sPAP and WHO FC were vital predictors for maternal prognosis.

Seven pregnant women (one in the pre-MDT group) with mitral valve stenosis underwent percutaneous balloon mitral valvuloplasty (PBMV) for severe lung congestion and dyspnoea before delivery. Subsequently, the symptoms were promptly relieved and a successful delivery occurred.

### Pulmonary-specific therapy for pregnancy with PAH

Not all pregnant women with PH underwent targeted drug therapy. Only patients with group 1 PH (e.g., idiopathic, CHD, connective tissue disease-associated PAH) received targeted drug therapy. If the increase in PAP was evaluated through echocardiography, a pulmonary vascular specialist would conduct PAH-specific therapy for continued pregnancy or women were hospitalized. Although the number of adequate, well-controlled studies of pulmonary-specific drugs for pregnant women is few, data from the retrospective literature suggests that pulmonary-specific therapy has improved maternal prognosis in pregnant PAH patients [15, 24]. The current PAH-specific drugs considered safe for the treatment of pregnant women consist of prostaglandin analogue (e.g., epoprostenol, treprostinil and iloprost) and phosphodiesterase 5 inhibitors (e.g. sildenafil and tadalafil), whereas endothelin receptor antagonists (e.g.ambrisentan, bosentan, and macitentan) are contraindicated during pregnancy for potential teratogenic effects [7, 12].

A Japanese study suggested that among the patients with higher pre-pregnant PAP, the mPAP increased with the progress of pregnancy [25]. Thus, pre-pregnant sPAP can be referenced for initiating PAH-specific therapy. In this study, the sPAP of six of eight pregnant women in the pre-MDT group who receiving PAH-specific therapy was > 60 mmHg on echocardiogram, and the sPAP was also > 60 mmHg in 19 of 22 women in the post-MDT group. Early use of pulmonary vasodilators has been found to prevent clinical worsening in non-pregnant patients with PAH [26, 27], and deterioration frequently occurs between weeks 20 and 24 [20]. Thus, our team usually recommends monotherapy for the control of PAP during the second trimester. In the perinatal period, if a pregnant woman has high-risk factors, the MDT will add prostaglandins or carry out early termination of pregnancy.

### Mode and timing of delivery

Caesarean section may be the advisable mode of delivery in pregnancy complicated by PH [9, 10, 20]. Obstetricians demonstrate a preference for caesarean section delivery for pregnant women with PH. Although five pregnant women who delivered via caesarean section died after delivery in the pre-MDT group, four of five deaths occurred due to severely unstable haemodynamics or obstetric indication. Moreover, after the implementation of the MDT, no deaths occurred in pregnant women who delivered via caesarean because of the regular follow-up and PAH-specific therapy, as well as meticulous postpartum care.

Our team realize that vaginal delivery is generally correlated with fewer cases of postpartum haemorrhage and infection in the general population; however, the haemodynamic and physiological variations during labour may easily induce RV failure in pregnant women with PH. Caesarean section is favoured by our obstetricians for the following possible reasons: (1) caesarean section avoids a further increase in CO and hemodynamic swings associated with labour; (2) planned caesarean section allows the MDT to develop a relatively optimal delivery plan (e.g., stabilisation of haemodynamics and evaluation of anaesthesia); (3) the maternal mortality risk of caesarean delivery may be higher after failed trials of labour [10].

The optimal timing of elective delivery in women with PH has been controversial. Although there was no significant difference in the median delivery time in the two groups, the post-MDT had a higher rate of preterm delivery (55.6% vs. 34.7%, p = 0.03). Most preterm births were discussed and determined by the MDT. Finally, although the premature birth rate has increased in the post-MDT group, many pregnant women are close to full-term delivery.

### Anaesthesia selection and challenge

Epidural anaesthesia is perhaps considered the preferred anaesthesia for PH pregnant women, while there has been rare robust evidence demonstrating that general anaesthesia significantly increases maternal mortality [28, 29]. Before the MDT was established, three patients died under general anaesthesia, and two died under epidural anaesthesia (n = 3/18, 16% vs. n = 2/20, 10%, *p* = 0.595), whereas no deaths occurred under planned caesarean section with general anaesthesia. In the post-MDT group, general anaesthesia was the major method used with invasive arterial blood pressure monitoring via a radial artery line, if available. In addition, the central venous pressure (CVP) of 70 patients was monitored, with no infection complications found. As revealed by the positive results, general anaesthesia may be a safe anaesthesia for pregnant women with PH with adequate preoperative preparation and intraoperative monitoring. General anaesthesia is capable of more effectively controlling the changes of patients’ condition during surgery; however, it is likely to affect the foetus. However, the time from anaesthesia to foetal delivery is usually very short, and our anaesthesiologists are adopting general anaesthesia more often.

### Limitations

Our analysis had several potential limitations. We recognise that randomised assignment to MDT or no-MDT is the gold-standard to measure the effects of the MDT. However, as that is not possible, we used a quasi-experimental interrupted time-series to conduct an observational study. Furthermore, this study was performed in a large tertiary pulmonary vascular disease centre and may not apply to community hospitals, where PH experts are not available. In addition, most data were collected from pregnant women with group 1 and group 2 PH, so our results might not reveal the effect of the MDT on other types of PH. Finally, our study is a single-center retrospective analysis and lacks uniform RHC, which may have caused statistical bias in the analysis. Therefore, multicenter and large sample research is needed.

## Conclusion

Implementing the MDT decreased the rate of urgent caesarean section and heart failure in patients with PH and no maternal deaths occurred in the post-MDT group. Pregnant women with severe PH and WHO FC III/IV might have a poor prognosis, whereas the use of pulmonary-specific therapy might improve outcomes of pregnancy in PH.

## Data Availability

The datasets used and/or analysed during the current study are available from the corresponding author on reasonable request.

## Abbreviations

ASD: Atrial septal defect
CHD: Congenital heart disease
CO: Cardiac output
LHD: Left heart disease
PAP: Pulmonary arterial pressure
PAH: Pulmonary arterial hypertension
PDA: Patent ductus arteriosus
PH: Pulmonary hypertension
RHC: Right heart catheterization
VSD: Ventricular septal defect
WHO FC: World Health Organization function class
WSPH: World Symposium on Pulmonary Hypertension
